# Nuclear translocation of the 4-pass transmembrane protein Tspan8

**DOI:** 10.1038/s41422-021-00522-9

**Published:** 2021-06-07

**Authors:** Yuwei Huang, Junjian Li, Wanqing Du, Siyang Li, Ying Li, Haozhi Qu, Jingxuan Xv, Li Yu, Rongxuan Zhu, Hongxia Wang

**Affiliations:** 1 State Key Laboratory of Membrane Biology, Tsinghua-Peking University Joint Center for Life Sciences, School of Life Sciences, Tsinghua University, Beijing, China; 2 State Key Laboratory of Oncogenes and Related Genes, Department of Oncology, Shanghai General Hospital, Shanghai Jiao Tong University School of Medicine, Shanghai, China

**Keywords:** Cell biology, Nuclear transport

Dear Editor,

Transmembrane proteins with multiple transmembrane domains are rarely found inside nuclei for good reason. To achieve intranuclear localization, the hypothetical transmembrane protein must first be extracted from its host membrane and the multiple hydrophobic transmembrane domains must be protected from the hydrophilic environment of the cytoplasm to maintain the proper conformation of the protein. Mechanistically, this poses considerable challenges.

Tetraspanin 8 (Tspan8) is a member of the tetraspanin family, all of which contain 4 transmembrane domains. Tetraspanin family proteins form lipid raft-like tetraspanin-enriched microdomains (TEMs) on the plasma membrane, which are highly enriched in cholesterol^1–6^. Tetraspanins contain multiple palmitoylation sites, and the palmitoylation of tetraspanins is required for its association with cholesterol^7–9^. By serendipity, we found that Tspan8-mCherry could localize inside nuclei. Z-stack live-cell imaging reveals that Tspan8-mCherry is present on the plasma membrane as expected; interestingly, a smeared Tspan8-mCherry signal is also present in the cytoplasm and inside the nucleus (Fig. 1a; Supplementary information, Video S1). HA-Tspan8 shows a similar localization pattern, which suggests that the position and type of tag do not affect the localization of Tspan8 (Supplementary information, Fig. S1a). In contrast, Tspan4, another tetraspanin family member, does not localize inside nuclei (Supplementary information, Fig. S1b). The nuclear localization of Tspan8 can be found in multiple cell lines, which suggests that it is a general phenomenon (Supplementary information, Fig. S1c). Importantly, endogenous Tspan8 is present in the nuclear fraction in multiple cell lines (Fig. 1b), suggesting that the nuclear localization of Tspan8 is not an artifact caused by overexpression.

**Fig. 1:**
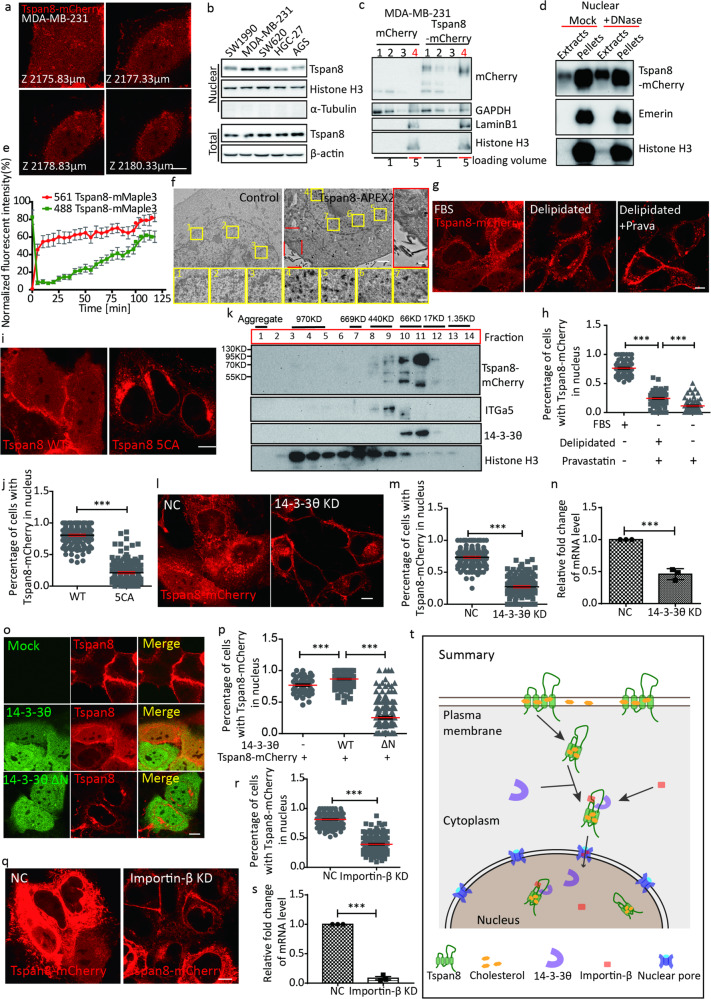
Nuclear translocation of the 4-pass transmembrane protein Tspan8. **a** MDA-MB-231 cells transfected with Tspan8-mCherry were observed by confocal microscopy. Z-stack images are shown. Scale bar, 10 µm. **b** Nuclear fractions and total cell lysates from different type of cells were collected. Endogenous Tspan8, Histone H3, α-Tubulin and β-actin were analyzed by western blot. **c** Nuclei from MDA-MB-231 cells expressing mCherry or Tspan8-mCherry were purified by density gradient centrifugation. Nuclei were harvested in fraction 4. Western blot was conducted to analyze the distribution of mCherry or Tspan8-mCherry. Loading volume ratio of fraction 1, 2 and 3 to fraction 4 is 1:5. **d** Nuclear pellet fractions collected as mentioned in Supplementary information, Fig. S2a were separated into two equal parts, one as control and the other digested by DNase for 30 min. After one more centrifugation, the soluble extracts and pellets were analyzed by western blot to detect Tspan8-mCherry, Emerin and Histone H3. **e** Statistical analysis of normalized fluorescence intensity in the cell nucleus. MDA-MB-231 cells overexpressing Tspan8-mMaple3 were used in a photo-conversion assay. The fluorescence intensities of the green and red versions of Tspan8-mMaple3 in the nucleus were traced and analyzed. Data are presented as means ± SEM. *n* = 15 from 3 independent experiments. **f** MDA-MB-231 cells expressing Tspan8-mCherry-APEX2 or WT cells (control) were fixed then subjected to DAB reaction and observed by TEM. Scale bar, 5 µm; zoom in, 2 µm. **g** Tspan8-mCherry-expressing MDA-MB-231 cells were cultured in medium with 10% fetal bovine serum (FBS), 10% delipidated serum or 10% delipidated serum with 30 µM Pravastatin for 36 h. Images were collected by confocal microscopy. Scale bar, 10 µm. **h** Quantification of the percentage of cells with Tspan8-mCherry in the nucleus from confocal images. Cells were cultured in variable media as mentioned in **g**. Data are presented as means ± SEM. *n* = 73 for FBS group, *n* = 83 for delipidated group and *n* = 77 for delipidated + Prava group from 3 independent experiments. ***P < 0.001, unpaired *t*-test. **i** MDA-MB-231 cells were transfected with WT Tspan8-mCherry or Tspan8-mCherry with mutations of all 5 palmitoylation sites (Tspan8 5CA). Cells were observed by confocal microscopy. Scale bar, 10 µm. **j** Statistical analysis of the percentage of cells expressing WT or mutant Tspan8 with Tspan8-mCherry in the nucleus from confocal images. Data are presented as means ± SEM. *n* = 147 for WT, *n* = 147 for 5CA from 3 independent experiments. ***P < 0.001, unpaired *t*-test. **k** A gel-filtration assay was performed on dialyzed nuclear extracts from Tspan8-mCherry-expressing MDA-MB-231 cells. Fractions of 1 ml were collected after gel-filtration. Tspan8-mCherry, Integrin α5 (ITGa5), 14-3-3θ and Histone H3 were analyzed by western blot. **l** MDA-MB-231 cells expressing Tspan8-mCherry (NC cells) or 14-3-3θ knockdown MDA-MB-231 cells expressing Tspan8-mCherry were imaged by confocal microscopy. Scale bar, 10 µm. **m** Quantification of the percentage of cells with nuclear Tspan8-mCherry in NC or 14-3-3θ knockdown cells from confocal images. Data are presented as means ± SEM. *n* = 142 for NC cells, *n* = 139 for 14-3-3θ knockdown cells from 3 independent experiments. ***P < 0.001, unpaired *t*-test. **n** Relative mRNA level of 14-3-3θ was verified by qPCR in NC and 14-3-3θ knockdown cells. Summary of 3 independent experiments. ***P < 0.001, unpaired *t*-test. **o** MDA-MB-231 cells expressing Tspan8-mCherry alone (top), with GFP-14-3-3θ (middle), or with GFP-14-3-3θ ∆N (bottom) were observed by confocal microscopy. Green indicates GFP signal; red indicates mCherry signal; yellow indicates merged signal. Scale bar, 10 µm. **p** Statistical analysis of the percentage of cells (expressing Tspan8-mCherry alone, Tspan8-mCherry + GFP-14-3-3θ, or Tspan8-mCherry + GFP-14-3-3θ ∆N) with Tspan8-mCherry in the nucleus from confocal images. Data are presented as means ± SEM. *n* = 57 for Tspan8-mCherry alone group, *n* = 140 for Tspan8-mCherry + GFP-14-3-3θ group and *n* = 163 for Tspan8-mCherry + GFP-14-3-3θ ∆N group from 3 independent experiments. ***P < 0.001, unpaired *t*-test. **q** NC or importin-β knockdown MDA-MB-231 cells expressing Tspan8-mCherry were observed by confocal microscopy. Scale bar, 10 µm. **r** Quantification of the percentage of cells (NC cells or importin-β knockdown cells) with Tspan8-mCherry in the nucleus. Data are presented as means ± SEM. *n* = 156 for NC cells, *n* = 154 for importin-β knockdown cells from 3 independent experiments. *** *P* < 0.001, unpaired *t*-test. **s** Relative mRNA level of Importin-β was verified by qPCR in NC and Importin-β knockdown cells. Summary of 3 independent experiments was shown. ***P < 0.001, unpaired *t*-test. **t** Diagram illustrating the translocation of Tspan8 into the nucleus.

Transmembrane proteins can be subject to protease cleavage, and the cytosolic cleavage product can translocate into the nucleus. Could the nuclear Tspan8-mCherry signal be a cleaved cytosolic fragment of Tspan8? If that is the case, we expect that the molecular weight (MW) of nuclear Tspan8-mCherry will be similar to the MW of mCherry, because Tspan8 only has ~7 amino acids on the C-terminal cytosolic side. Instead, we find that nuclear Tspan8-mCherry has a similar MW to cytosolic Tspan8-mCherry (Fig. 1c). To determine whether Tspan8-mCherry is localized on the nuclear membrane or in the nucleoplasm, we subjected cells to hypotonic lysis, then separated the nuclear fraction into the pellet fraction, which contains the nuclear membrane and chromatin, and the soluble nucleoplasm fraction. The nucleoplasm fraction does not contain the nuclear membrane protein Emerin, which indicates that this fraction is not significantly contaminated with nuclear membrane. Under these conditions, we observe a small amount of Tspan8-mCherry in the nucleoplasm (Supplementary information, Fig. S2a). Similarly, the S100 fractions from nuclei and cytosol also contain Tspan8-mCherry, which suggests the presence of membrane-free Tspan8-mCherry (Supplementary information, Fig. S2b). Treating the nuclear preparation with DNase significantly enhanced the amount of Tspan8-mCherry, but not Emerin, in the nucleoplasm (Fig. 1d). This suggests that Tspan8-mCherry may be trapped in chromatin.

To test whether cytosolic Tspan8-mCherry can translocate into nuclei, we tagged Tspan8 with mMaple3, a photo-convertible fluorescent protein. We find that shortly after we photo-converted Tspan8-mMaple3 in the cytoplasm, the converted Tspan8-mMaple3 signal starts to rise in the nucleus, which suggests that cytosolic Tspan8 can enter nuclei (Fig. 1e; Supplementary information, Fig. S2c). Next, we investigated in which form Tspan8 translocate into nuclei. We reasoned that since Tspan8 contains 4 transmembrane domains, the most likely scenario would be that Tspan8 is translocated into the nucleus in the form of vesicles. In this way, the 4 transmembrane domains can be protected by membrane to maintain the right conformation. To test this hypothesis, we carried out APEX2-based intracellular specific protein imaging by electron microscopy^10^. APEX2 catalyzes the local deposition of diaminobenzidine, which binds osmium, thus enhancing the contrast in TEM images. First, we confirmed that adding the APEX2 tag at the C-terminus of Tspan8-mCherry did not affect nuclear translocation of Tspan8-mCherry (Supplementary information, Fig. S2d). The enhanced contrast can be observed on retraction fibers, where Tspan8 is enriched, which suggests that APEX2 labeling works properly. To our surprise, we find that the APEX2 signal inside the nucleus is not associated with any membrane structure (Fig. 1f). This indicates that Tspan8-mCherry-APEX2 does not translocate into nuclei in the form of vesicles.

How does Tspan8 maintain its conformation without a membrane to protect its hydrophobic transmembrane domains? Since tetraspanins can bind cholesterol, is it possible that Tspan8 is transported as a Tspan8/cholesterol complex? In this scenario, the hydrophobic transmembrane domain can be protected by cholesterol, thus maintaining the correct conformation. To test this hypothesis, we depleted the cholesterol by culturing cells in cholesterol-free medium (with delipidated serum) in the presence of a cholesterol synthesis inhibitor (Pravastatin). We found that 36 h after cholesterol depletion, the cholesterol level was moderately reduced (Supplementary information, Fig. S2e). Strikingly, almost all the nuclear Tspan8 disappears after cholesterol depletion; instead, most of the Tspan8 is localized on the plasma membrane (Fig. 1g, h). Although cholesterol depletion has complicated and profound effects on cells, this result does consistent with the hypothesis that cholesterol is directly or indirectly required for nuclear translocation of Tspan8. As mentioned above, palmitoylation of tetraspanins is required for association with cholesterol. Therefore, we generated a mutant Tspan8 in which all 5 putative palmitoylation sites were mutated. We find that the nuclear localization of mutant Tspan8 is significantly reduced (Fig. 1i, j). Together, these data suggest that association with cholesterol is directly or indirectly required for nuclear translocation of Tspan8. This hypothetical Tspan8**-**cholesterol complex may exist as a TEM, which is a nanometer-sized lipid**-**protein assemblage; alternatively, it may exist as a single Tspan8 molecule bound to cholesterol. To test the assembly status of Tspan8 in the nucleus, we carried out gel-filtration analysis of nuclear Tspan8. We find that although there is a small amount of nuclear Tspan8 present in the high MW fraction, the vast majority of nuclear Tspan8 is present in the low MW fraction (Fig. 1k). This suggests that Tspan8 is not likely transported into the nucleus as TEM.

14-3-3 proteins are known for their roles in regulating the subcellular localization of their client proteins. 14-3-3 proteins can promote the cytosolic localization of some clients while promoting the nuclear localization of others. We find that R18, a 14-3-3 peptide inhibitor, blocks the translocation of Tspan8 into nuclei (Supplementary information, Fig. S3a, b). In this study, we used MDA-MB-231 cells, which highly express 14-3-3θ and 14-3-3 ζ. We find that knockdown of 14-3-3θ, but not 14-3-3ζ (Supplementary information, Fig.S3c–e), blocks nuclear translocation of Tspan8 (Fig. 1l–n). Similarly, overexpressing the dominant negative mutant of 14-3-3θ (with an N-terminal deletion of amino acids 1**–**127) also blocks Tspan8 nuclear translocation (Fig. 1o, p). Consistent with this, we find that 14-3-3θ binds to Tspan8 in nuclei (Supplementary information, Fig. S3f). These data suggest that 14-3-3θ is required for translocation of Tspan8 into nuclei. Importin-β is a nucleocytoplasmic transport receptor that transports proteins and RNAs cross the nuclear pores^11,12^. We find that importin-β is required for nuclear translocation of Tspan8 (Fig. 1q–s). Put together, these data suggest that Tspan8 is transported into nuclei using the canonical nuclear transportation pathway for cytosolic proteins.

In summary, our study suggests that the 4-pass transmembrane protein Tspan8 can achieve nuclear localization by using the machinery for nuclear translocation of cytosolic proteins. Our data suggest that cholesterol is important for this nuclear translocation, possibly by binding and protecting the hydrophobic transmembrane domains during the translocation process (Fig. 1t). Our study opens up the possibility that multiple transmembrane proteins can traffic into nuclei for functions that are not recognized currently.

## Supplementary information

Supplementary Figures

Supplementary Video S1
