# Supplementary material for: Nuclear translocation of the 4-pass transmembrane protein Tspan8
Source: Cell Res. 2021 Jun 7;31(11):1218–21. doi: 10.1038/s41422-021-00522-9 (PMC8563794; doi:10.1038/s41422-021-00522-9)
Supplement: Supplementary file 1 — Supplementary Figures [file 41422_2021_522_MOESM1_ESM.pdf]

Materials and methods

Plasmids and cell culture

Plasmid construction

cDNA of Tspan8 was cloned into pmCherry-N_1 for imaging and biochemistry experiments. cDNA of Tspan8 was cloned into pcDNA3.1 tagged with 3xFlag for Co-IP experiments. WT 14-3-3θ and 14-3-3θ ΔN (128-245) cDNAs were cloned into pEGFP-C_3.

Tspan8 palmitoylation mutant (Tspan8 5CA)

At all 5 sites, the cysteine residue for attachment of the palmitoyl chain was changed to alanine by site-directed mutagenesis of Tspan8-mCherry. Changes in the Tspan8 5CA mutant were confirmed by sequencing.

Cell lines

MDA-MB-231 cells, SW1990 cells and their derivatives, were cultured at 37°C and 5% CO_2 in DMEM supplemented with 10% serum and 1% penicillin-streptomycin. H1975, HCC827, PC9, B16 and 4T1 cells were cultured at 37°C and 5% CO_2 in RPMI-1640 supplemented with 10% serum and 1% penicillin-streptomycin.

Cell transfection

For MDA-MB-231 and all the other cells, a 3.5 cm-dish of cells (~1×10^5) were transfected with 2.5 μg DNA using a Lipofectamine-3000 transfection kit (Invitrogen) and then grown for 24 h for protein expression.

Gene knockdown by siRNA

SiRNA design and transfection

SiRNAs were all ordered from Invitrogen. The siRNA sequence for knocking down 14-3-3θ was CCGACAAGAAGTTGCAGCTGATTAA. The siRNA sequence for knocking down importin-β was CAGTCTGGCTGAAGCTGCTTATGAA. SiRNAs were transfected into MDA-MB-231 cells using a Lipofectamine-3000 transfection kit. The cells were then grown for 48 h for further experiments.

Knockdown efficiency assessment

Total RNA was extracted from cells using Trizol reagent (Invitrogen), and 1 μg RNA was used as a template for reverse transcription with random primers. qPCR was performed to characterize the mRNA levels of specific genes using 2 × RealStar Green Power Mixture (GeneStar) in a Roche Light 480 Real-Time PCR machine. Primer sequences for 14-3-3θ were: forward 5'-GTGGTGATGATCGAAAACAAAC-3'; reverse 5'-ATGATGAGGGTGCTGTCTTT-3'. Primer sequences for importin-β were: forward 5'-TCCAGATACGAGGGTACGAG-3'; reverse 5'-TCGCATAAAACTTGCTGGTG-3'.

Cell Nuclear purification and detection

Quick nuclear purification for nuclear protein detection

A 10-cm dish of cells (~1× 10^7) were collected and washed in PBS. The cell nucleus was purified by ExKine™ Nuclei Extraction Kit (KTP4002). Nuclear protein samples and total cell lysate samples were separated and detected by western blot assay respectively.

Antibody information

All antibodies were purchased from commercial companies, anti-mCherry antibody (Takara, 632496), anti-Tspan8 antibody (Abcam, ab70007), anti-GAPDH antibody (Proteintech, 60004-1), anti-Histone H3 antibody (CST, 4499s), anti-LaminB1 antibody (Proteintech, 12987-1), anti- α-Tubulin antibody (MBL, PM054), anti-β-actin antibody (Zen BioScience, 2000688F10), anti-ITGa5 antibody (CST, 4705s), anti-Flag antibody (Sigma, F1804), anti-Emerin antibody (CST, 30853T) and anti-14-3-3θ antibody (CST, 9638s).

Cholesterol depletion assay

MDA-MB-231 cells were pre-cultured with 10% FBS, 10% delipidated serum (Tecono F810) or 10% delipidated serum plus 30 μM Pravastatin (TargetMol, T0672) for 12 h. Then the cells were transfected with Tspan8-mCherry plasmid and cultured in the same medium as before for a further 24 h. At the end of the experiment (36 h in total), the cholesterol level of cells was measured by Amplex® Red cholesterol assay kit (Invitrogen, A12216) according to the manufacturer's instructions.

Nuclear extract preparation

MDA-MB-231 cells expressing Tspan8-mCherry were collected from ten 10-cm culture dishes and washed in PBS. The cell pellet was resuspended in 3× volume of A buffer (10 mM Tris-HCl (pH = 7.9), 1.5 mM $MgCl_2$, 10 mM KCl and freshly added 1 mM DTT) on ice for 10 min with cocktail inhibitors (Roche) and then further lysed by a Dounce tissue grinder (~7 strokes). Next, after 12000 rpm centrifugation for 15 min at 4°C, the suspension and pellet were collected respectively, the suspension as cytosol (stored on ice for further experiments) and the pellet as rough nucleus. Then the pellet was

homogenized in 0.5 volume of B buffer (20 mM Tris-HCl (pH = 7.9), 25% glycerol, 1.5 mM $MgCl_2$, 0.2 mM EDTA (pH = 8) and 20 mM KCl). Then the same volume of C buffer (20 mM Tris-HCl (pH = 7.9), 25% glycerol, 1.5 mM $MgCl_2$, 0.2 mM EDTA (pH = 8) and 1.2 M KCl) was dropped into the system, and the mixture was gently rotated at 4°C for 30 min. After 10000rpm centrifugation for 30 min at 4°C, the suspension was collected as nuclear extract and the pellet was collected as nuclear pellet.

S100 fraction preparation

0.11× volume of 10×S100 buffer (300 mM Tris-HCl (pH = 7.9), 30 mM $MgCl_2$ and 1.4 M KCl) were added into the cytosol and the nuclear extract generated during nuclear extrat preparation process, respectively. Then the samples were applied to 100000g ultracentrifugation at 4°C for 1 h. Finally, the S100 and P100 fractions were collected and then for western blot analysis.

Gel-filtration assay

Dialysis of nuclear extract

The nuclear extract was dialyzed in 10× volume of D buffer (20 mM Tris-HCl (pH = 7.9), 20% glycerol, 0.2 mM EDTA (pH = 8), 100 mM KCl and freshly added 1 mM DTT) for 4 h. The final product was dialyzed nuclear extract.

Gel-filtration

The chromatographic column (Superose6 Increase 10/300 GL) was pre-equilibrated with more than 30 ml of D buffer. The dialyzed nuclear extract was injected into the column, and separated samples (1 ml each) were collected in tubes. The gel-filtration samples were separated by SDS-PAGE and the proteins were detected by western blot.

Photo-conversion experiments

Photo-conversion experiments were conducted using a NIKON A1 confocal microscope, fitted with a 60× oil objective and 3 times zoom in. The area of interest on the bottom of the cell was photo-converted by 405 nm laser with 10% output for 1 s to partially convert Tspan8-mMaple3 from original green (excited, 488nm laser; emission, ~507nm) to red (excited, 561nm laser; emission, ~610nm). Immediately after conversion, the focal plane was changed from the bottom of the cell to the nucleus. Then 488 and 561 nm lasers were applied for imaging and each field of 512 × 512 pixels was imaged for a total of 2 h with intervals of 5 min. Finally, the total fluorescent signals in nucleus were traced and analyzed by use of NIS-analysis software.

Diaminobenzidine (DAB) staining and preparation for TEM imaging

MDA-MB-231 cells transfected with Tspan8-mCherry-APEX2 or not (as negative control) were cultured on 3.5-cm dishes overnight. Transfected cells were fixed using room temperature 2.5% glutaraldehyde in APEX reaction buffer (100 mM sodium cacodylate with 2 mM $CaCl_2$, pH 7.4), then quickly moved onto ice for 45 min. Fixed cells were rinsed 5 times in chilled buffer for 2 min, then incubated for 5 min in buffer containing 20 mM glycine to quench unreacted glutaraldehyde, followed by 5× 2 min rinses in chilled buffer. A freshly diluted solution of DAB (1 mg/ml, Sigma) was applied to the cells for 2 min, then 0.003~0.03% (v/v) H_2O_2 was added into the system at room temperature for 1~15 min depending on the sample. Finally, the cells were rinsed for 5× 2 min in chilled buffer. To prepare the cells for TEM imaging, they were rinsed 3 times in distilled water and incubated in 2% aqueous uranyl acetate (Electron Microscopy Sciences) overnight. Then the samples were dehydrated in a graded ethanol series (50%, 70%, 80%, 90%,

100%, 100%) for 2 min each on ice, and finally incubated in 100% ethanol for 2 min at room temperature. The cells were then infiltrated and embedded in SPI-Pon 812 resin. The interesting areas of DAB staining were identified and cut out using a razor blade and mounted on resin blocks with cyanoacrylic adhesive. Finally, trimmed blocks were cut into 70-nm ultrathin sections for examination by TEM (Hitachi, H7650).

Imaging

Confocal microscopy imaging was conducted with an Olympus FV1200 microscope, fitted with a 60× oil objective. Picture size was 1024 × 1024 pixels. Z-stack images were captured using a step size of 0.5 μm from the cell bottom to the cell surface.

Statistics

All experiments were conducted independently at least three times.

P values were calculated with the two-tailed unpaired t-test using GraphPad Prism5. Error bars, mean ± SEM. *$P \leq 0.1$, **$P \leq 0.01$, ***$P \leq 0.001$, ns, non-significant.

Supplementary Information, Fig S1

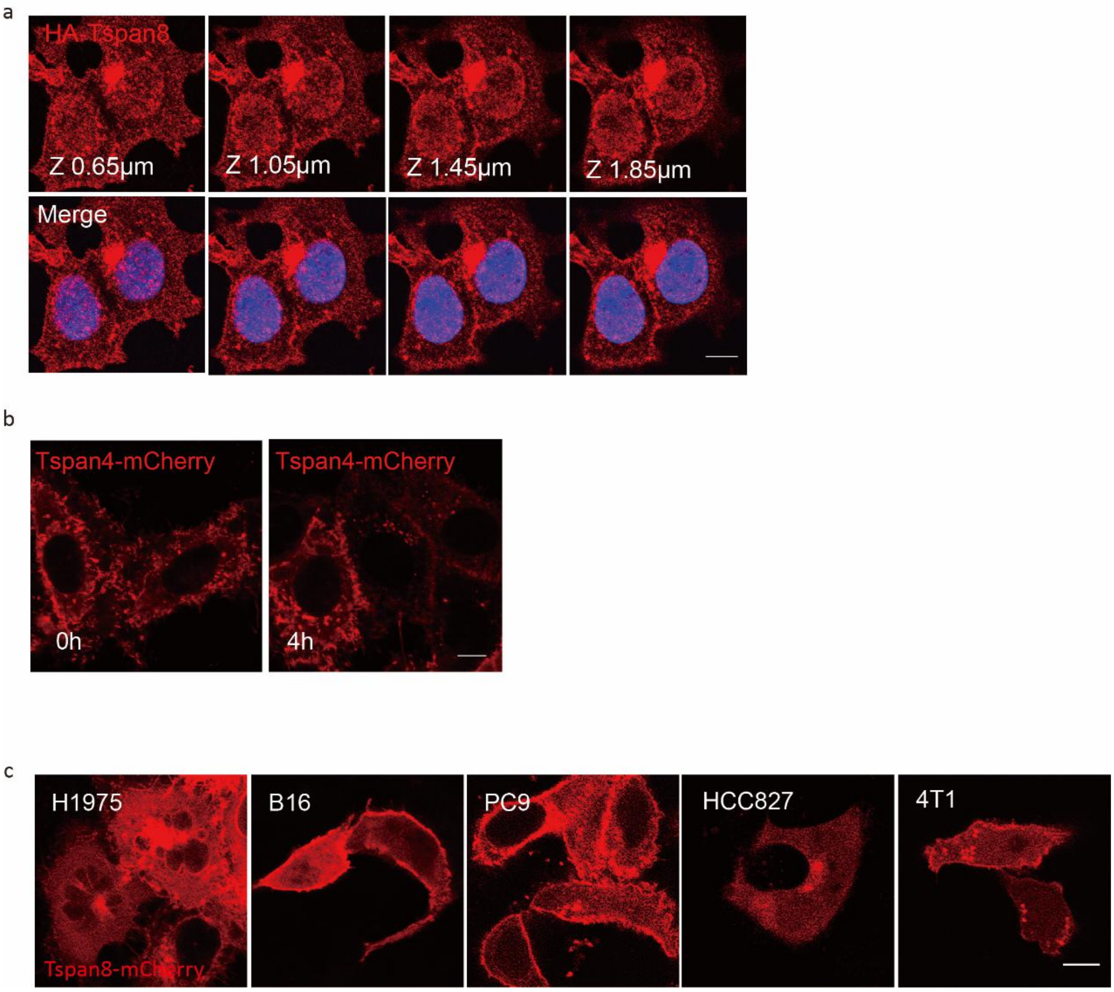

a. Immuno-fluorescent analysis of SW1990 cells expressing HA-Tspan8. HA antibody was used to detect HA-Tspan8. Nuclei were stained with DAPI. Z-stack images were taken by confocal microscopy. Red, HA-Tspan8; blue, DAPI. Scale bar, 10 μm.

b. MDA-MB-231 cells expressing Tspan4-mCherry were imaged by confocal microscopy. Scale bar, 10 μm.

c. Different types of cancer cells, including H1975, B16, PC9, HCC827 and 4T1, were transfected with Tspan8-mCherry and observed by confocal microscopy. Scale bar, 10 μm.

Supplementary Information, Fig S2

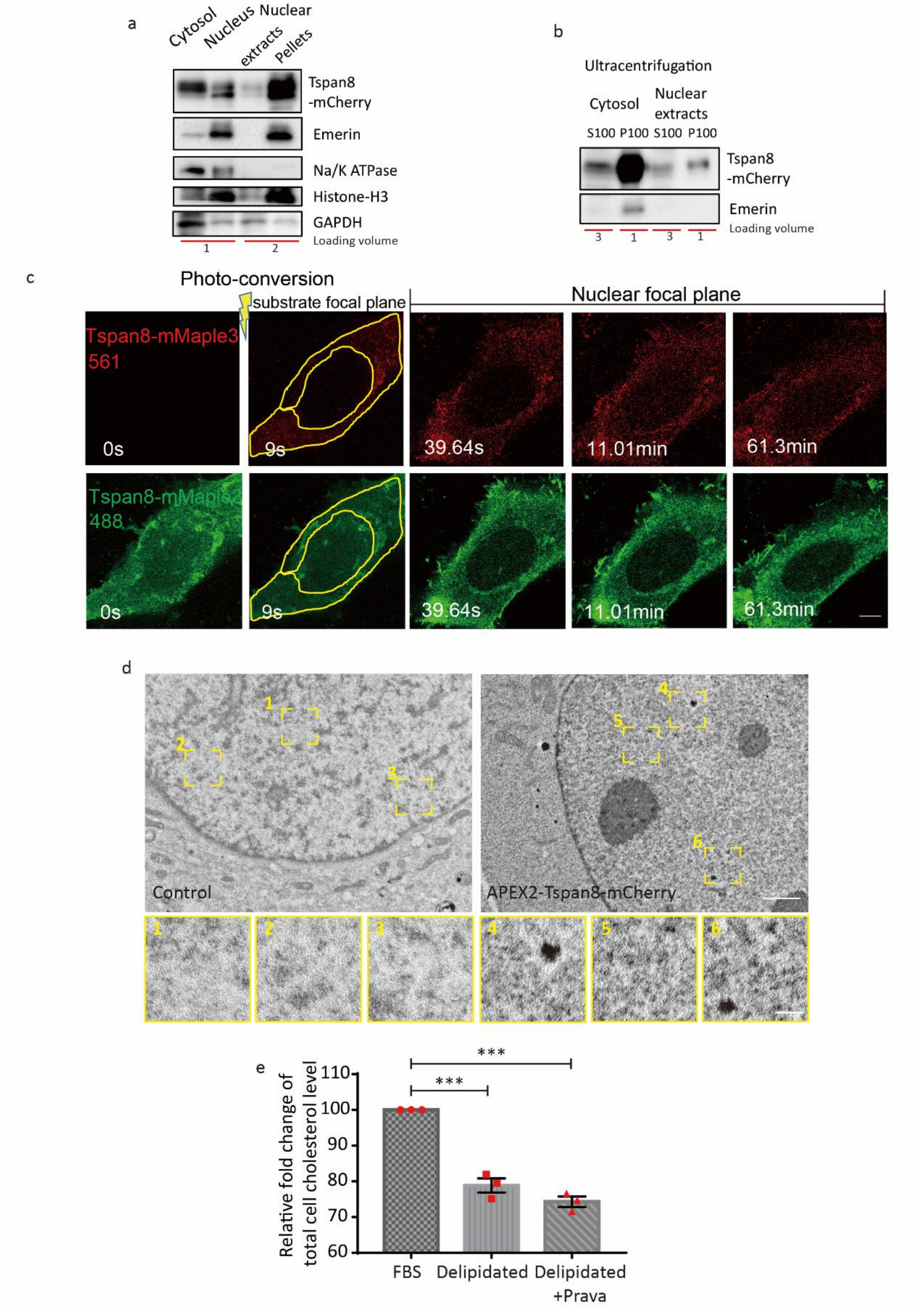

a. Nuclear and cytosol fractions were purified from MDA-MB-231 cells expressing Tspan8-mCherry. The nuclear fractions were further separated into soluble extracts and pellets. A western blot assay was performed to detect the levels of

Tspan8-mCherry, Emerin, Na/K ATPase, Histone H3 and GAPDH proteins. The loading volume ratio of the different fractions are shown at the bottom.

b. Cytosol and nuclear extracts from MDA-MB-231 cells expressing Tspan8-mCherry were subjected to ultracentrifugation to collect S100 (suspension) and P100 (pellet) fractions. A western blot assay was then conducted to detect Tspan8-mCherry. The loading volume ratio of the different fractions are shown at the bottom.

c. Time-lapse imaging of the photo-conversion assay in MDA-MB-231 cells expressing Tspan8-mMaple3. Photo-converting laser was focused on the bottom of the cell (the photo-converting area was indicated by yellow line), and the Tspan8-mMaple3 fluorescent signal was monitored in the nuclear focal plane. Scale bar, 10 μm.

d. MDA-MB-231 cells expressing APEX2-Tspan8-mCherry or WT cells (control) were fixed then subjected to DAB reaction and observed by TEM. Scale bar, 5 μm; zoom in, 2 μm.

e. Relative total cellular cholesterol level of cells cultured with 10% fetal bovine serum (FBS), 10% delipidated serum or 10% delipidated serum with 30 μM Pravastatin for 36h. Data shown represent mean ± SD. n = 3 independent experiments, ***$P < 0.001$.

Supplementary Information, Fig S3

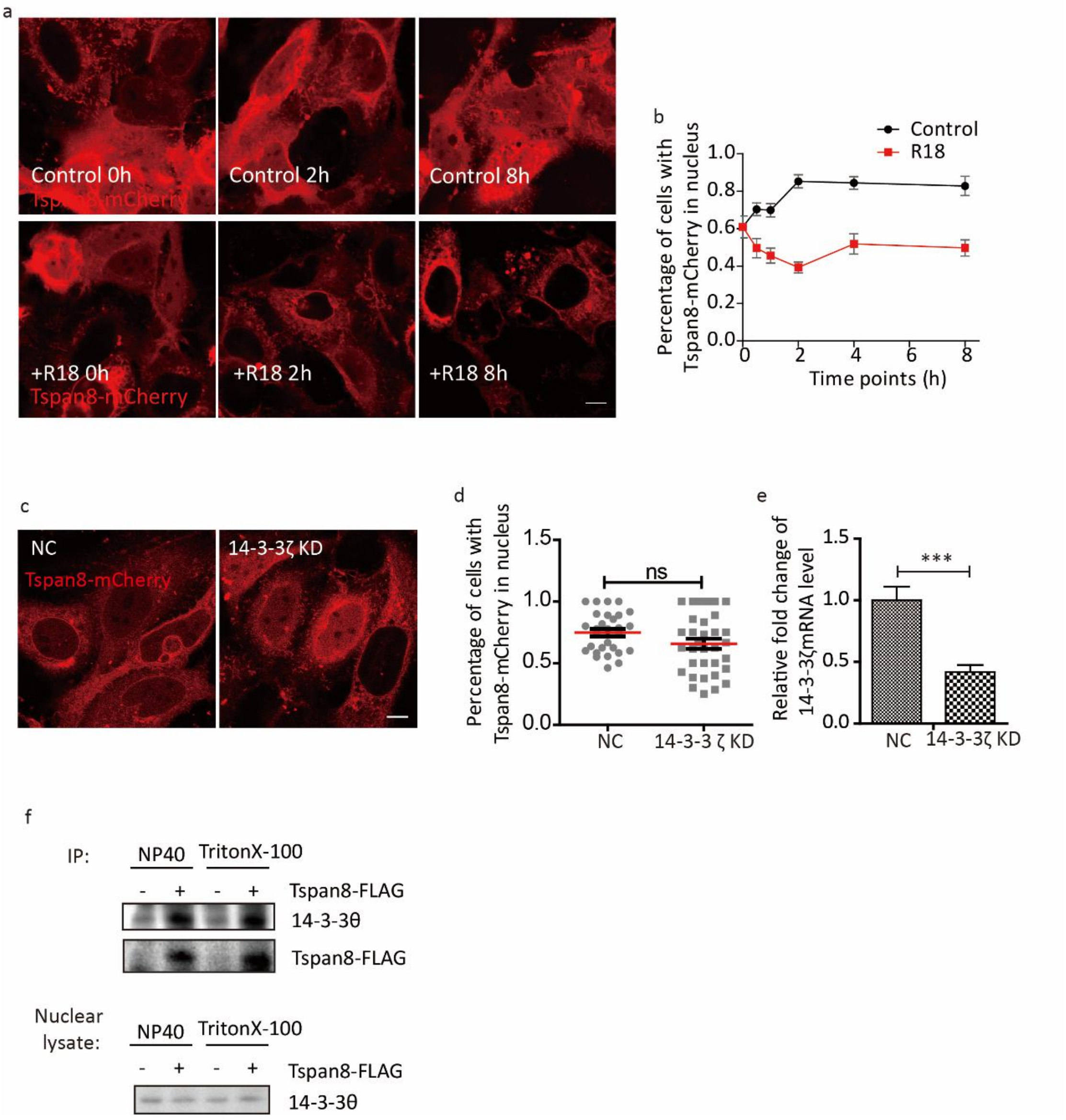

a. MDA-MB-231 cells expressing Tspan8-mCherry were treated without (control) or with the 14-3-3 peptide inhibitor R18 (20 μM), then imaged at 0 h, 2 h and 8 h by confocal microscopy. Scale bar, 10 μm.

b. Quantification of the percentage of cells with Tspan8-mCherry in the nucleus from confocal images. Cells were treated without or with R18 as mentioned in a.

c. NC or 14-3-3ζ knockdown MDA-MB-231 cells expressing Tspan8-mCherry were

observed by confocal microscopy. Scale bar, 10 μm.

d. Quantification of the percentage of cells (NC cells or 14-3-3ζ knockdown cells) with Tspan8-mCherry in the nucleus. Data are presented as means ± SEM. $n = 29$ for NC cells, $n = 34$ for 14-3-3ζ knockdown cells, which is the representative experiment of 3 independent experiments. NS= non-significant, unpaired *t*-test.

e. Relative mRNA level of 14-3-3ζ was verified by qPCR in NC and 14-3-3ζ knockdown cells. Summary of 3 independent experiments was shown. $^{***}P < 0.001$, unpaired *t*-test.

f. A co-immunoprecipitation assay was conducted on purified nuclei from MDA-MB-231 cells expressing Tspan8-Flag. Anti-Flag antibody was used to precipitate Tspan8-Flag. The levels of 14-3-3θ and Tspan8-Flag proteins were detected by western blot. Loading controls are shown in the lower nuclear lysate panel.

Supplementary Information, Video 1

Movie of MDA-MB-231 cells expressing Tspan8-mcherry at nuclear focal plane conducted by confocal microscopy. Scale bar, 5μm.
